# Enhanced Multimodal Brain Tumor Classification in MR Images using 2D ResNet as backbone with Explicit Tumor Size Information

**DOI:** 10.7150/jca.95987

**Published:** 2024-06-11

**Authors:** Yunhao Zeng, Nianbo Liu, Xinduoji Yang, Chenke Huang, Ming Liu

**Affiliations:** 1 School of Information and Software Engineering, University of Electronic Science and Technology of China, Chengdu 611731, China.; 2 Yangtze Delta Region Institute (Quzhou), University of Electronic Science and Technology of China, Quzhou 324000, China.; 3 Quzhou People's Hospital, Quzhou Affiliated Hospital of Wenzhou Medical University, Quzhou 324000, China.

**Keywords:** Tumor Size, Gliomas, Convolutional Neural Networks, Multimodality

## Abstract

It's a major public health problem of global concern that malignant gliomas tend to grow rapidly and infiltrate surrounding tissues. Accurate grading of the tumor can determine the degree of malignancy to formulate the best treatment plan, which can eliminate the tumor or limit widespread metastasis of the tumor, saving the patient's life and improving their prognosis. To more accurately predict the grading of gliomas, we proposed a novel method of combining the advantages of 2D and 3D Convolutional Neural Networks for tumor grading by multimodality on Magnetic Resonance Imaging. The core of the innovation lies in our combination of tumor 3D information extracted from multimodal data with those obtained from a 2D ResNet50 architecture. It solves both the lack of temporal-spatial information provided by 3D imaging in 2D convolutional neural networks and avoids more noise from too much information in 3D convolutional neural networks, which causes serious overfitting problems. Incorporating explicit tumor 3D information, such as tumor volume and surface area, enhances the grading model's performance and addresses the limitations of both approaches. By fusing information from multiple modalities, the model achieves a more precise and accurate characterization of tumors. The model I s trained and evaluated using two publicly available brain glioma datasets, achieving an AUC of 0.9684 on the validation set. The model's interpretability is enhanced through heatmaps, which highlight the tumor region. The proposed method holds promise for clinical application in tumor grading and contributes to the field of medical diagnostics for prediction.

## 1. Introduction

The World Health Organization (WHO) defines glioma as a tumor that arises from the brain's neuroglial cells 1. It is the most common primary intracranial tumor 2, mainly appearing in the hemispheres of the brain (66.8%) and cerebellum (8.6%) 3. The annual incidence rate of glioma in China is 5-8 per 100,000 people, and the 5-year mortality rated the third following pancreatic cancer and lung cancer among systemic tumors, according to the China Glioma Guidelines (2022 Edition) 4. Symptoms caused by gliomas vary depending on their location and size, and the main clinical manifestations include three major categories: increased intracranial pressure, neurological and cognitive disorders, and epileptic seizures 5,6. Malignant gliomas tend to grow rapidly and infiltrate surrounding tissues, posing serious threats to patients' health 7,8. Treatments for gliomas include surgical resection, radiotherapy, chemotherapy, etc. 9. Unfortunately, the prognosis for patients is not satisfactory. Consistent follow-up monitoring is also essential due to the significant risk of recurrence and metastasis in gliomas 10,11. The fifth edition of the WHO Classification of Tumors of the Central Nervous System classifies gliomas into four grades (I-IV), with low-grade gliomas (grades I and II) and high-grade gliomas (grades III and IV) 1. Most low-grade gliomas will eventually evolve into high-grade gliomas 12. Accurate grading of the tumor can determine the degree of malignancy to formulate the best treatment plan. Appropriate treatments can eliminate the tumor or limit widespread metastasis and prevent further progression of the tumor, saving the patient's life and improving their prognosis.

Due to the potential secondary harm caused by biopsy, the grading of gliomas currently favors the use of non-invasive imaging methods, including magnetic resonance imaging (MRI). MRI can reveal the diffusion of lesions at the interface between the inner and outer boundaries of tumor blood vessels or the inner and outer walls of tumor cell interstitial tissues, as well as the distribution of blood flow, providing high-resolution spatial imaging information 13. Combing with deep learning technology, multi-modal MRI has revolutionized medical imaging in recent years. Multi-modal MRI data provides multiple complementary pieces of information for the tumor microenvironment, such as tumor morphology, cell density, and blood flow 14,15. The various T1 and T2 values of different tissues in the human body make it possible to generate images. Water has a low signal on T1WI and darker grey matter, whereas water has a high signal on T2WI and brighter grey matter 16,17. Different MRI sequences carry diverse information, with free water suppressed and bound water having a higher signal in images obtained by inversion recovery sequences. Furthermore, contrast medium like gadolinium can enhance the signal intensity of magnetic resonance and affect the T1 and T2 relaxation of surrounding protons 18, reflecting different tumor information. In this study, we utilize deep learning algorithms to automatically extract useful features from large-scale multi-modal MRI data, which serve as key factors for tumor identification and grading.

Currently, 2D convolutional neural networks (CNNs) are commonly employed for tumor grading in the field of medical imaging. Previous studies often use diverse strategies to integrate data from various modalities through multiple networks or channels, aiming to achieve multi-channel-based multi-modal feature extraction 19-21. In contrast, a 3D image is a three-dimensional data structure composed of stacked 2D images. It effectively represents the tumor's volume, spatial location, and temporal information among the 2D images, leading to improved results in other studies that leverage 3D CNN 22-24. However, the increased complexity of 3D imaging data can hinder model training, potentially resulting in not only training instability and increased computational costs, but also failure to achieve the desired outcomes in tumor detection and grading.

It has been pointed out that volumetric analysis of brain tumors is a decisive factor in brain tumor detection 25, and that the calculation of tumor volume also has significant numerical advantages in terms of tumor size, region, and treatment 26. Inspired by this, we incorporate the most important 3D features of gliomas (e.g., tumor volume, surface area), instead of whole 3D images, into the grading model, which provides a novel and effective approach utilizing multi-modal MRI to leverage the benefits of both 2D and 3D features. Using Pyradiomics, we extract 14 3D features from tumor images.

Additionally, we adapt the ResNet50 architecture to predict glioma grading by integrating these features with those obtained by the 2D CNN at the fully connected layer. The segmented image containing only the tumor is obtained, and the four modalities (T1, T2, T1-Gd, and FLAIR) are overlaid on each of the four channels of the image, serving as inputs to the model. The experimental results demonstrate that the inclusion of 3D features in our approach leads to an improvement of approximately 1.2% over the multi-modal 2D CNN, yielding optimal results and confirming the method's efficacy in this study and its potential for clinical medicine.

For 2D network structure, 3D information of the tumor is concealed and does not stand out prominently among the numerous image features. We are the first to discover the significant contribution of these 3D features to tumor grading. This study aims to develop and validate a tumor grading model using the multi-modal MRI and the 3D features of the tumor, based on the 2D ResNet network. Then, we conduct a comparative analysis of our proposed method through separate experiments using 2D CNN and 3D CNN. Additionally, the interpretability of the model is given by heatmaps.

## 2. Materials and Methods

### 2.1 Datasets

Two publicly available datasets for brain gliomas are used in this study: the UCSF-PDGM dataset and the BraTS2020 dataset. The UCSF-PDGM dataset 27 comprises 495 adult patients diagnosed with histologically confirmed grade II-IV diffuse gliomas, consisting of 56 grade II, 43 grade III, and 396 grade IV tumors. The dataset includes 199 females and 296 males, with a mean age of approximately 57 years and an average overall survival of approximately 574 days from the initial diagnosis to the last clinical follow-up. Cranial stripping is performed on the dataset using publicly available deep learning algorithms and 295 grade IV tumors are included in the BraTS2021 Challenge. The entire dataset undergoes automatic segmentation using an ensemble model that incorporates the winning segmentation algorithms from the BraTS Challenge. Subsequently, the images are manually corrected by trained radiologists and reviewed by 2 expert reviewers for final approval. In addition to T1, T2, T1-Gd and FLAIR modalities, the dataset also provides five other modalities, including SWI, HARDI, etc. The tumor data in the BraTS Challenge originates from 19 distinct medical centers. The primary objective of the competition is to foster the advancement of medical image segmentation techniques and facilitate the provision of enhanced tools for brain tumor diagnosis and treatment. The BraTS2020 competition, held in 2020, includes a training set comprising 369 cases, featuring an average age of approximately 61 years and an average total survival of about 446 days. The dataset encompasses tumor grading information, segmentation labels, as well as NIFTI files for four modalities per case. A series of preprocessing operations are conducted on the data from both datasets to create a more balanced grading ratio within the dataset.

### 2.2 Data Preprocessing

Following the WHO definition, we reclassify the UCSF-PDGM dataset into a binary dataset consisting of low-grade gliomas (LGG) and high-grade gliomas (HGG). We also incorporate the cases categorized as LGG in BraTS2020, with each case containing four modalities (T1, T2, T1-Gd and FLAIR) and a corresponding tumor segmentation map. Each case is represented as 3D data in NIfTI format with dimensions of 240x240x155. Initially, we convert each case into 155 PNG images of size 240x240. Then, we extract the tumor region based on the tumor segmentation map. As a result, the new dataset comprises a total of 112 LGG cases and 407 HGG cases.

**Figure 1:**
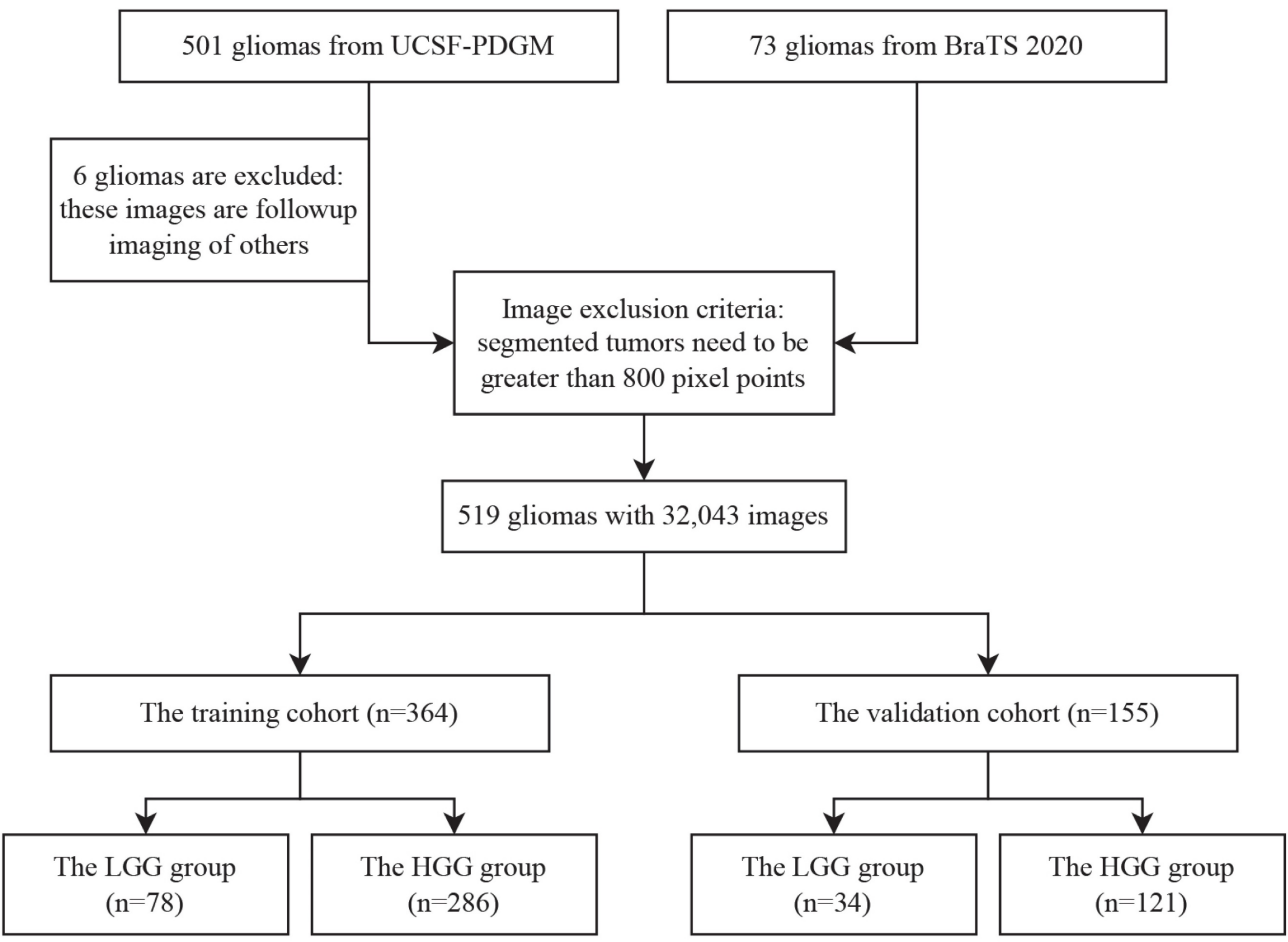
Data division illustrating the inclusion and exclusion criteria. A total of 55 gliomas are excluded based on tumor size or follow-up imaging, and the remaining cases are divided into training and validation cohorts.

Subsequently, we partition the dataset into training and validation sets based on patients, with a ratio of 7:3. The training set comprises 78 LGGs and 286 HGGs, while the validation set contains 34 LGGs and 121 HGGs. However, due to the significant difference in the number of LGGs and HGGs, we perform up-sampling to address the long-tailed distribution issue. Additionally, we employ data augmentation techniques exclusively for LGGs, resulting in a final input of 25,162 images in the training set and 6,881 images in the validation set, as shown in **Figure 1**.

Our proposed method involves incorporating the intrinsic 3D features of the images into the features obtained from training a 2D CNN. To extract these 3D radiomic features, we utilize the Pyradiomics package (v3.0.1). Pyradiomics can extract various radiomic features from images such as Gray Level Co-occurrence Matrix (GLCM), 2D Shape and 3D Shape features. Ultimately, for each case, we choose only 14 easily accessible 3D shape features extracted by Pyradiomics, including Mesh Volume, Voxel Volume, Surface Area, Surface Volume Ratio, Sphericity, Maximum 3D Diameter, Maximum 2D Diameter Slice, Maximum 2D Diameter Column, Maximum 2D Diameter Row, Major Axis Length, Minor Axis Length, Least Axis Length, Elongation and Flatness.

### 2.3 Data Augmentation

Compared to the original image size of 240x240, even after excluding cases that still have tumors appearing too small. For LGG images in the training set, we first resize the images to 448x448. Then, we perform a central crop to obtain images of size 224x224. Finally, we randomly crop them to 160x160. For HGG images in the training set and the validation set, we directly perform a central crop to obtain images of size 160x160. A study has shown that flipping techniques can effectively capture features in medical images, generating more discriminative feature maps 28. Following the recommendations from this study 22, we also incorporate horizontal and vertical flipping into our data augmentation process. Additionally, we apply CyclicShift 29, a method that moves the image in a random direction and cyclically fills the out-of-box parts to the other side. This technique helps to avoid losing original image pixels while preserving their semantic information as much as possible. It also forces the model to effectively utilize local and fragmented information. Furthermore, we include random rotation in the range of [-180°, 180°] to prevent overfitting and enrich the data patterns of replicated images. At last, we use salt and pepper noise, which introduces noticeable noise and distortion in certain regions of the image. This encourages the model to better learn the semantic information of the target and helps the model become more robust and resilient.

### 2.4 Model

In the selection of the tumor grading model, we consider that a few 2D images of a patient may not capture the complete information of the entire tumor. By employing a 2D CNN, the tumor is projected into a single planar image, resulting in the loss of temporal spatial information. This absence of information adversely affects the accuracy of tumor grading. Conversely, while the utilization of 3D CNN allows for the direct consideration of the complete tumor information, excessive information introduces additional noise, thereby impeding model training and leading to a severe overfitting issue. Additionally, the 3D model demands more computational resources due to its higher computational intensity. In a study aiming to improve the expected lifespan of brain tumor patients, tumor grading was performed through volume analysis 30. To overcome the limitations of both 2D CNN and 3D CNN, we propose a novel approach for tumor grading that combines 2D CNN with 3D features. This fusion of 2D and 3D information enhances the grading performance of the model while mitigating resource consumption.

We adopt the standard ResNet50 network as the backbone of our model. This choice ensures that the model has stable and reliable performance while enabling the use of pre-trained parameters. The residual blocks in the ResNet consist of one or more convolutional layers and a shortcut connection that directly adds the input of the unit to its output. This design allows deep networks to be trained more easily without losing information, addressing the issues of gradient vanishing and exploding.

In MRI, different imaging modalities provide different information. T1WI highlights the transverse relaxation differences in tissues, providing clearer visualization of the tissue structure of the brain and other organs, reflecting global information. On the other hand, T2WI emphasizes the longitudinal relaxation differences in tissues, offering a more intuitive display of lesions. Additionally, different tissues such as white matter, bone marrow, cerebrospinal fluid, and fat exhibit varying signal intensities across different imaging sequences, allowing the model to learn different feature maps in different modalities. The fusion of information from multiple modalities can result in a more precise and accurate characterization and understanding of tumors by removing redundant information and complementing missing information.

**Figure 2:**
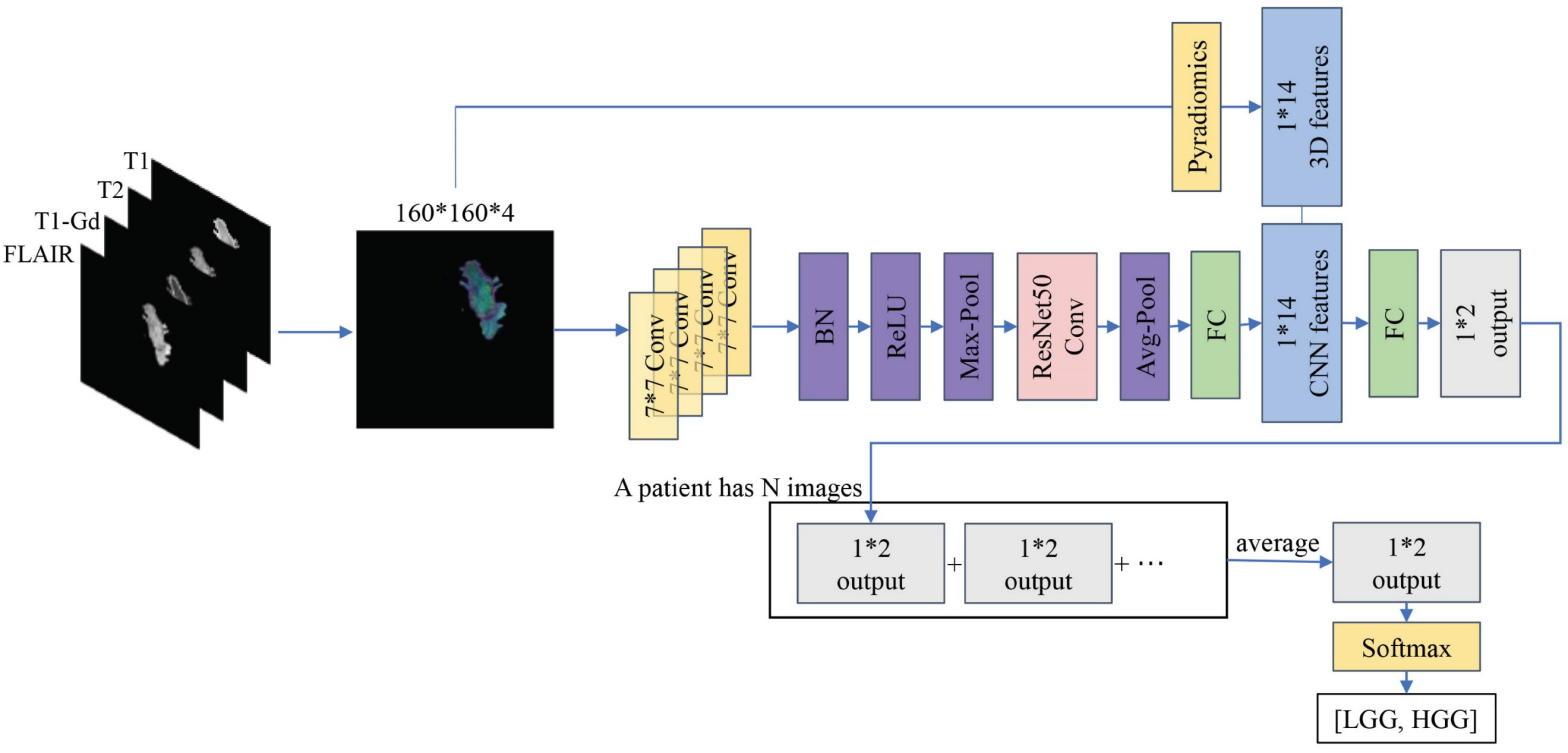
The workflow of the proposed method. The left side indicates that four modalities are superimposed on four different channels to constitute multimodal data. On the one hand, the features are extracted by 2D CNN with ResNet50 as the backbone, on the other hand, 14 3D features are extracted by using Pyradiomics, and both of them are superimposed to output a 1*2 vector. The lower illustration shows that a patient with multiple 2D MRIs can obtain multiple 1*2 vectors, which are then combined to generate graded prediction results.

Based on this, we integrate the data from four modalities, namely T1, T2, T1-Gd and FLAIR, into the network. Considering that the model needs to process this information simultaneously without separate computations, in this study, the data from the four modalities are combined via channel stacking. This approach ensures that the model can simultaneously observe multi-modal information while reducing computational costs. Unlike previous networks, the model proposed in this study expands its output from a single channel to four channels, constituting the 2D information of the images. During data preprocessing, 14 3D radiomic features are extracted for each patient's tumor. Recognizing the equal importance of the features learned by the CNN model and the radiomic features, we modify the fully connected layer of ResNet50. The learned feature vectors are first scaled to 14 values, and each tumor image is combined with the corresponding 14 3D radiomic features of the patient. Finally, these 28 features are directly outputted as a 1x2 vector. We apply Softmax to the output vector, ensuring that the sum of the probabilities of the image being LGG and HGG is 1. In each step, the model learns the information from a batch of images and adjusts the parameters through an optimizer. At the end of an epoch, we obtain the 1x2 outputs for all the images. Finally, we reclassify these vectors according to patients, and the average of many 1x2 output vectors for each patient becomes the final prediction of our model for the tumor grading of that patient. The architecture of our model is shown in **Figure 2**.

For model training, we set 20 epochs with a batch size of 16 for the training set. We use the Adam optimizer with a learning rate of 0.001. The L2 regularization weight decay is set to 5e-4. The gradient decay rate is 0.9 and the squared gradient decay rate is set to 0.99. The loss function used is cross-entropy. All experiments in this study are conducted using Python 3.9.16, PyTorch 2.0.0, and two NVIDIA GeForce GM200 graphics cards with CUDA 12.0 installed.

### 2.5 Statistical analysis

The ROC curve is a graphical tool used to illustrate the performance of a classification model. It plots the true positive rate (TPR) against the false positive rate (FPR) at different thresholds. We choose AUC (Area Under the Curve) as the primary evaluation metric in this study. The AUC represents the area under the ROC curve and is one of the most important metrics used in the medical field to assess the performance of binary classifiers. A higher AUC value indicates better classifier performance.

Since the proportion of LGG to HGG in the validation set is close to 1:4, using accuracy as an evaluation metric will result in a misleading interpretation. If all tumors are classified as HGG, the accuracy (Acc) would still reach 78%, making it unsuitable for comparing the model's classification ability. Therefore, we adopt the average per-class accuracy (APCA) alongside accuracy to evaluate the model's strengths and weaknesses in this study. APCA calculates the accuracy for each class and then averages them to obtain the final result.



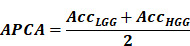



Finally, we use sensitivity (SEN) and specificity (SPE) as metrics for evaluating the accuracy of the model on samples from different categories.

## 3. Results

### 3.1 The proposed model

**Figure 3:**
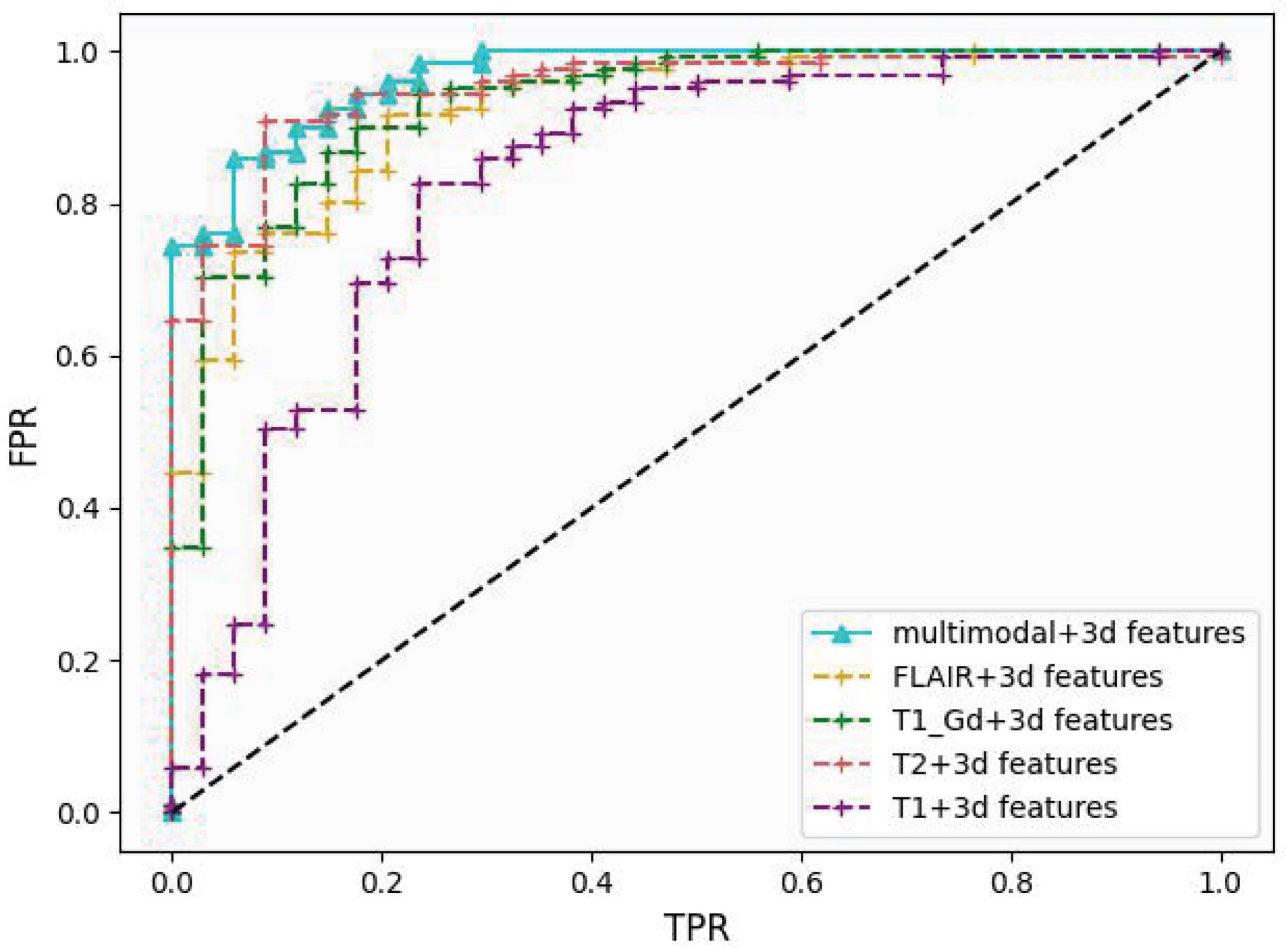
Performance comparison of different models on the validation cohort using ROC curves. Our method's curve (blue solid line) achieves the highest score at nearly all reference points.

Our proposed model utilizes ResNet50 as the backbone and data from the four modalities (T1, T2, T1-Gd and FLAIR) is fed to the model to extract the required key features. In order to integrate the 3D features better, we modify the infrastructure of ResNet50 by replacing the inputs from multiple channels to multiple pathway concatenation. With these improvements, our model achieves an AUC of 0.9684 on the validation set, providing strong evidence of its efficacy in extracting multi-modal features, as shown in **Figure 3**.

**Figure 4:**
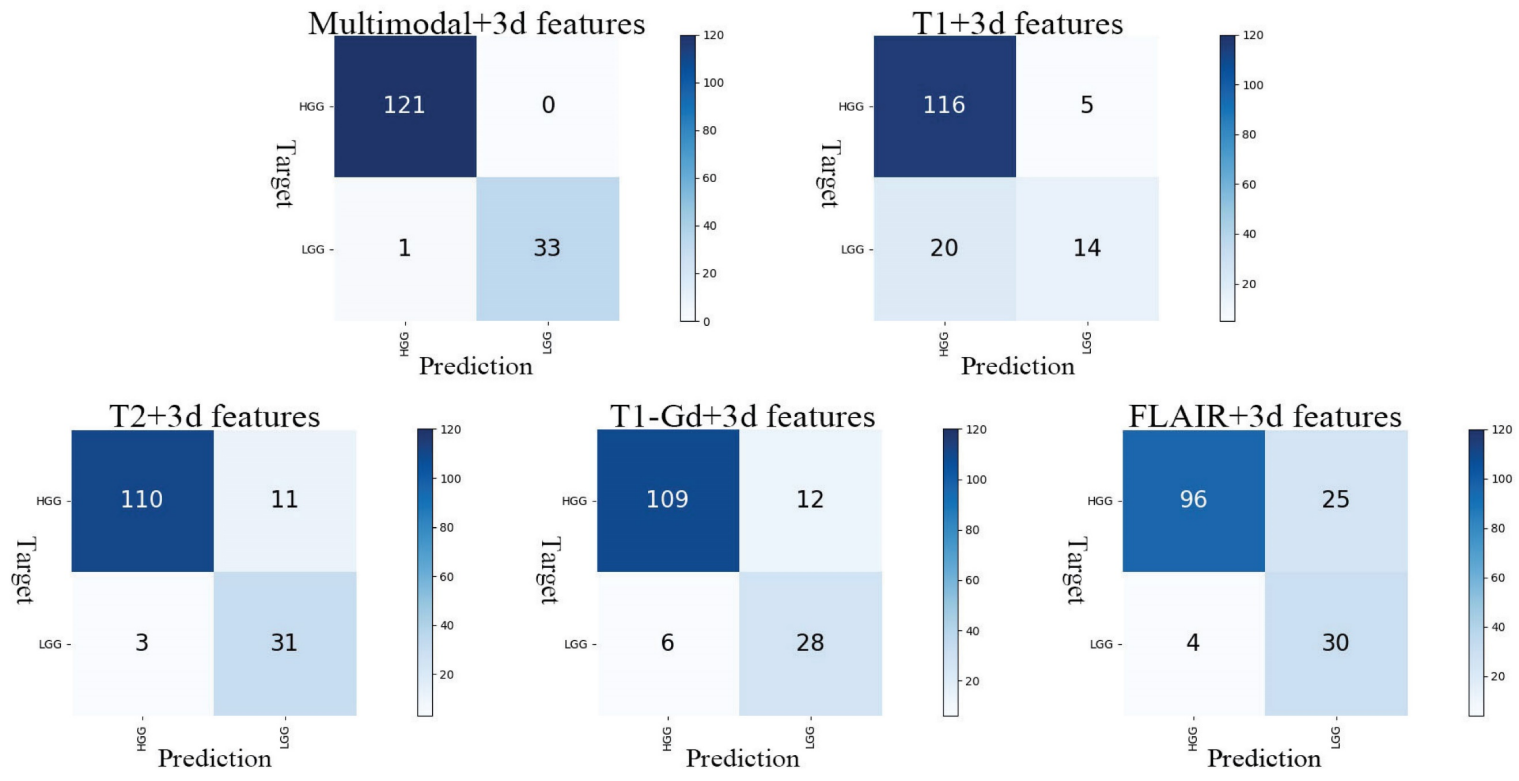
Confusion matrix for the five models. The y-axis represents the actual grading, and the x-axis represents the predicted grading.

In our optimal result, Acc is 0.9936, APCA is 0.9853, SEN is 1.0000 and SPE is 0.9706. Observation of the model's confusion matrix reveals that only one case of LGG was not correctly classified, as shown in **Figure 4**.

### 3.2 Multi-modal and compared with 2D

To validate the ability of the multi-modal approach to learn more information and improve the model's tumor grading capability, we conduct experiments by applying 2D CNN with 3D features separately to each modality. Apart from changing the input of ResNet50 to a single channel, all other parameters remain unchanged. The results are shown in **Table 1**. The AUC obtained from the T1 modality is the lowest at 0.8284, while the highest AUC is achieved with the T2 modality at 0.9499. The results of the single-modality experiments are lower than the AUC obtained with the multi-modal approach, indicating that the multi-modal method significantly enhances the model's ability to grade tumors. This confirms the effectiveness of training with combined multi-modalities.

Subsequently, we conduct a control experiment using only 2D CNN without incorporating 3D features. In this experiment, the multi-modal images, composed of the four modalities, are fed into the ResNet50 model. The fully connected layer directly outputs a 1x2 vector representing the model's classification of the tumor as LGG or HGG. The data augmentation method and model parameters remain unchanged. This experiment achieves an AUC of 0.9565 on the validation set. Based on these findings, we conduct separate experiments for each single modality using 2D CNN alone and the results obtained from each modality on 2D CNN are used as the baseline for that modality.

The results in **Table 1** demonstrate that our method improves the performance by 18.3% for T1, 3.2% for T2, 2.0% for T1-Gd, and 1.5% for FLAIR. In the experiment with the combination of all four modalities as input, there is a 1.2% improvement. Furthermore, the experiments conducted with 2D CNN reveal that multimodality consistently outperforms individual modalities in terms of AUC. These experiments demonstrate the effectiveness of our proposed approach of 2D CNN with 3D features and incorporating multiple modalities in the tumor grading task.

### 3.3 Compared with 3D

We further implement a ResNet50 model based on 3D CNN to compare the results of directly using the 3D model with our proposed method. We choose the same training and validation sets used in the 2D CNN experiment and apply additional data augmentation techniques to the LGG samples in the training set. However, we make slight modifications to the data preprocessing and augmentation methods. First, we convert each patient's DICOM files into 155x240x240 2D images containing only the tumor. Next, we select center cropping, horizontal flipping, vertical flipping and random rotation at four angles as the data augmentation methods in order to preserve the original 3D information of the tumor. Also, we use a global variable to apply the same data augmentation to all images from a particular patient and select the middle 128 images. At last, the remaining 2D images are concatenated to form a 3D image of size 128x160x160, which serves as the input. Similarly, we conduct experiments using T1, T2, T1-Gd, FLAIR and the combination of all four modalities, resulting in a total of five 3D CNN experiments. We choose the 3D ResNet50 model with a batch size of 4 while keeping the other parameters unchanged.

The results of the 3D experiments are shown in **Table 2**. The AUC obtained from the multi-modal 3D CNN is only 0.6626, significantly lower than the AUC achieved with our proposed method of combining 2D and 3D features. This demonstrates the effectiveness of our method. However, regardless of the modality, the results of 3D CNN are lower than those of 2D CNN used alone. Additionally, the AUC obtained from the multi-modal approach is lower than the values obtained from the single modalities.

### 3.4 Ablation study

Finally, we conduct ablation experiments on the data augmentation methods used in 2D+3d_features, as shown in **Table 3**. When we remove the CyclicShift method from the data augmentation, the AUC decreases by approximately 1.0%. This indicates that the presence of CyclicShift helps the model focus more on local features, thereby improving the tumor grading capability of the model. Additionally, the rotation and flipping data augmentation methods have been proven effective in the tumor grading model. In the experiments without data augmentation, we only apply center cropping to the training and validation sets to ensure consistent input size. The results in **Table 3** demonstrate that the use of data augmentation and replication strategies significantly improves the AUC.

### 3.5 Explainability

**Figure 5:**
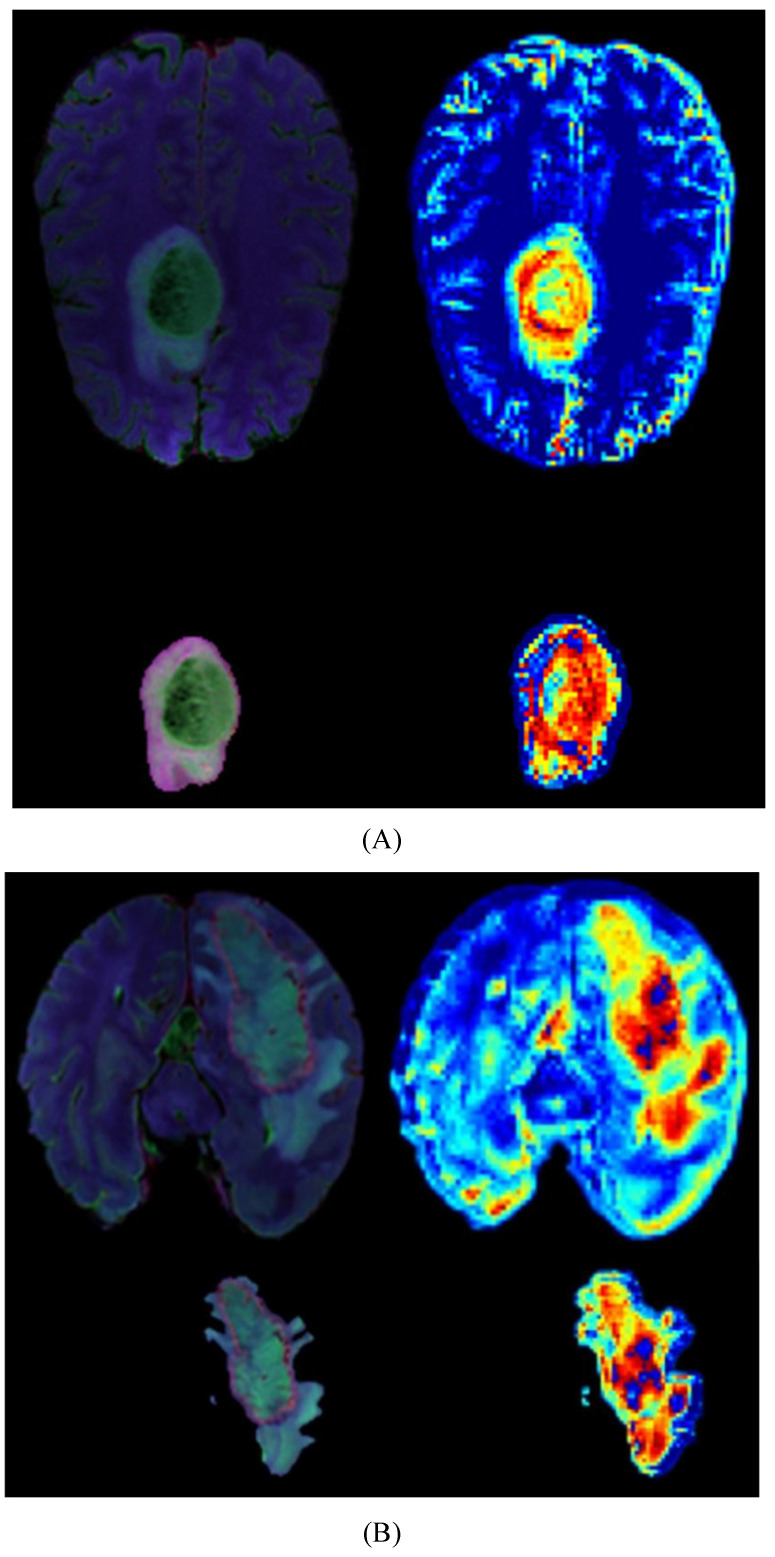
Heatmaps for LGG(A) and HGG(B), each map includes a whole-brain map and a tumor map with four modal overlays. The darker red regions indicate the areas where the model focuses most.

We provide model explainability through heatmaps, as shown in **Figure 5**. The darker red regions indicate the areas the model focuses on the most. In the original images, the model primarily focuses on the glioma in the brain. Based on the image segmentation results, we retain only the tumor regions as input for the model. The segmented images include necrotic regions, edema areas, and enhanced tumor regions. The heatmaps generated from these segmented images provide a more detailed focus area, and we can observe that the deeper red regions on the tumor correspond to the most prominent areas learned by the model during tumor grading.

### 3.6 Survival analysis

**Figure 6:**
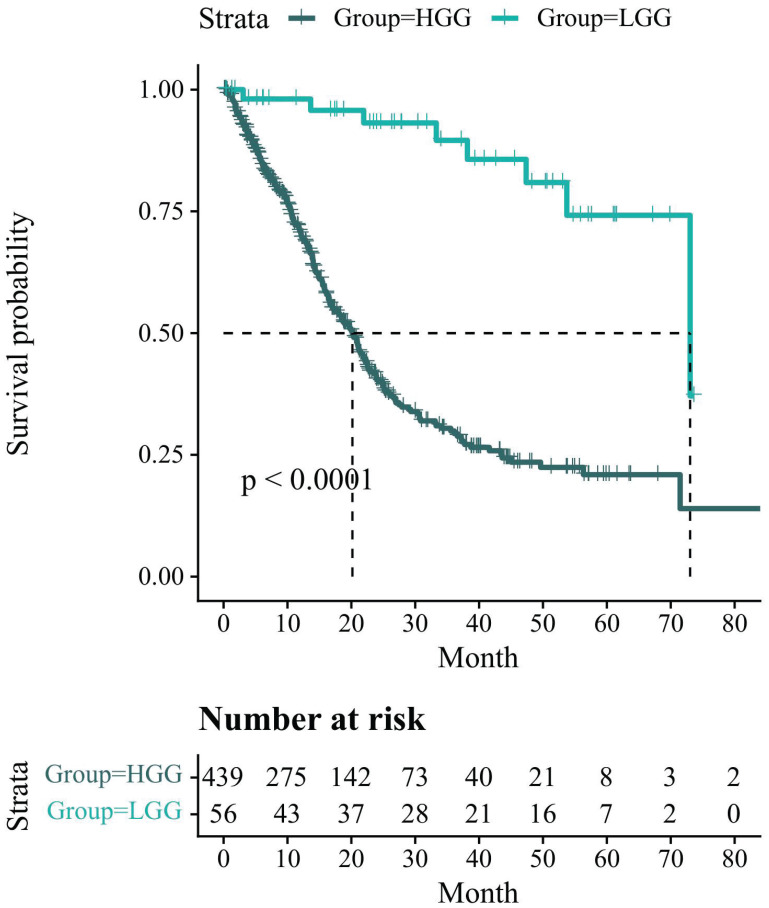
Kaplan-Meier survival analysis information related to the glioma status in the UCSF-PDFM patient cohort. The survival probability of patients is represented on the y-axis and the follow-up time is represented on the x-axis.

**Figure 6** presents the Kaplan-Meier survival analysis information related to the glioma status in the UCSF-PDFM patient cohort. However, there is a missing value for HGG. To compensate for this, we remove the top and bottom 10 patients in HGG after sorting by overall survival (OS) and replace the missing value with the average. All survival times are reported in months. The results indicate that the overall survival probability of LGG patients is higher than that of HGG patients and the number of HGG patients who survive decreases more rapidly. After conducting the log-rank test, we find that p<0.0001<0.05, indicating that the difference in survival status between the two groups cannot be explained by sampling error alone. The grouping factor is the reason for the divergence in survival rates between the two curves.

## 4. Discussion

Currently, the choice between 2D and 3D CNN for glioma grading tasks in multi-modal MRI primarily depends on the characteristics of the dataset and the requirements of the task. 2D CNN is mainly used for processing slice images in MRI, allowing for convolution and feature extraction on a two-dimensional plane. It independently processes the images on each slice and then combines the features of the slices for grading. In contrast, 3D CNN is suitable for handling combinations of multiple MRI slices or a sequence. This model can perform convolution and feature extraction in three directions (width, height, and depth), capturing spatial information and temporal relationships in volumetric data. Compared to 2D CNN, 3D CNN can better utilize the correlation between slices. However, due to its more complex structure, it consumes more computational resources.

For 2D CNN, the differences among various studies mainly lie in the selection of slices. František Šefčík *et al.* 31 designed a simple 4-layer 2D CNN model and used the interpretability of the model's output as an additional training objective, combined with the original classification objective, to train the neural network model. During training, they stacked three modalities (T1, T2, and FLAIR) and selected only the slice with the largest tumor as input. In a study involving 161 patients, Jialin Ding *et al.* 21 achieved the highest AUC of 0.8980 by developing a method that combines radiomics and 2D CNN only on the T1 modality. They selected 851 extracted radiomic features and utilized inputs from the section with the largest tumor area along with two adjacent images. In another study involving 1166 patients, Yoo Seong Choi *et al.* 32 achieved a fully automated process that included segmentation and grading. In the grading stage, they used two modalities (T2 and T1-Gd) and a tumor mask. They selected five MRI slices of each patient as input for the 2D CNN. The aforementioned three papers chose either the slice with the largest tumor itself or included surrounding parts of the image but failed to provide comprehensive tumor information. In a study by Ruyi Qu *et al.* 19, which focused on tumor methylation, they directly inputted all 2D images containing tumors for each patient and trained separate models for four modalities (T1, T2, T1-Gd and FLAIR). They combined the extracted features using self-attention mechanisms and used LSTM to determine the grading category. Soumick Chatterjee *et al.* 20 created a category for images without tumors, converting the grading task into a three-class classification. The input consisted of the four modalities (T1, T2, T1-Gd and FLAIR) stacked 2D images. Although both papers include all images containing tumors as input, misclassification can easily occur when the tumor size is too small.

For 3D CNN, Hiba Mzoughi *et al.* 22 proposed a 3D CNN framework for automatic brain glioma classification using the entire volume of the T1-Gd sequence. When compared to 2D CNN, it generated more discriminative feature maps and achieved an accuracy of 96.49% on the validation set. Sérgio Pereira *et al.* 24 compared the differences between inputting whole-brain images and tumor regions. They conducted experiments on four modalities (T1, T2, T1-Gd and FLAIR) and analyzed the results using heatmaps. They found that using tumor ROI for prediction yielded better results, with the highest accuracy reaching 92.98%. In a study involving 470 patients, Chenan Xu *et al.* 23 developed a fully automated segmentation and grading model based on 3D CNN. They used three multi-modalities (T1, T2 and T1-Gd) and combined the features extracted from 3D CNN with tumor shape, texture, and other radiomic features. Finally, they inputted the combined features into an SVM for classification, achieving an AUC of 0.9580.

We refer to the strategy employed in the aforementioned papers to train our model and combine the advantages of 2D and 3D approaches to propose a method for glioma grading that utilizes both 2D CNN and 3D features. Additionally, we design comparative experiments between 2D CNN and 3D CNN. The experimental results presented in **Table 1** and **Table 2** indicate that the method using 2D CNN with 3D features achieves the optimal AUC of 0.9684 when combining the four modalities. Compared to using only 2D or 3D CNN, our method shows improvements in both single-modality and multi-modality (from 0.9565 to 0.9684). Since the T1 sequence focuses more on global features, when we input images with tumors only for this modality, the performance of the 2D CNN-based grading is poor, with an AUC of only 0.6451, which is lower than the result of 0.6631 obtained by 3D CNN. However, our method significantly improves the performance of the ResNet50 model for glioma grading in the T1 modality by incorporating 3D features, reaching an AUC of 0.8284. The addition of 3D features provides the missing temporal information to the 2D CNN model, and the combination of these features contributes to accurate grading.

Furthermore, from the tables, we also observe that multi-modality achieves higher SEN compared to single-modality. In the 2D CNN experiments, the SEN for multi-modality reaches 0.9811, while our proposed method achieves 1.000, indicating that the features learned through multi-modality are advantageous in distinguishing classes with more diverse distributions. Next, by observing the SPE, we can see that after incorporating 3D features, both single-modal and multi-modal experiments show a significant improvement in SPE. In the case of multi-modality, the SPE increases from 0.6531 to 0.9706, suggesting that our method can alleviate the impact of class imbalance on the model.

Based on our experimental results, except for the T1 sequence, the AUC for the other three modalities and the multi-modal 3D CNN experiments are lower than the 2D CNN results. Additionally, the multi-modal 3D CNN results are lower than the results of the other four single modalities. We speculate that when the convolutional layers transition from 2D to 3D, the learned feature maps become more complex. When multiple feature maps are learned simultaneously through multi-modality, various feature maps influence the judgments of the 3D CNN, resulting in a lower AUC.

During the data augmentation process, due to the adoption of random cropping and CyclicShift, the tumors inputted to the model may be segmented into two parts or not be complete. However, considering that in actual diagnosis, doctors also observe multiple 2D images and tumor images obtained from different directions may not be complete either, it is reasonable that the model should possess the ability to accurately grade incomplete tumors. In terms of the model's running speed and cost-effectiveness, we conduct a comparison using a 3D model consisting of four modalities. In this 3D model, we employ two GPUs with a combined graphics memory of 22,658 MB, resulting in a processing time of approximately 5 minutes and 30 seconds per round. Conversely, our proposed model, utilizing a mere 1,965 MB of graphics memory, exhibits a significantly reduced processing time of about 4 minutes and 10 seconds per round. These findings demonstrate that our model not only lowers the configuration requirements but also achieves a higher AUC at a faster pace, thereby presenting distinct advantages in the context of clinical practice.

The limitations of this study mainly lie in the following aspects. Firstly, the data used in this study comes from two datasets comprising multiple medical centers. Although the same exclusion criteria are applied, there may be differences in the imaging scanners used for imaging due to the different periods. The extent to which these discrepancies might impact glioma grading remains a subject of controversy and necessitates further standardization efforts. Also, although 519 cases have been included in our experiment, more data should be added for future experiments to meet the diversity of patients. Secondly, when combining the features extracted by CNN and the 3D features, we assume that the importance of these two types of features is equal. Then, we compress the features extracted by CNN through fully connected layers to 14 features. However, it is worth noting that this compression process and the presumed equal significance of the features might introduce potential biases into the grading results. To address this limitation, we plan to use reinforcement learning to automatically select the optimal number of features in each training round to obtain more accurate predictive grading results.

## 5. Conclusion

In this study, we develop and validate a multi-modal model for grading prediction of gliomas by using ResNet50 as a backbone and combining 2D CNN with explicit tumor 3D information. Extensive experiments demonstrate that our model achieves the state-of-the-art AUC of 0.9684 on the validation set. Furthermore, we verify that the model can focus on the tumor region through visualization. Therefore, using only the image of the tumor as input can make the model more able to focus on the information present in the tumor. In conclusion, our proposed method accurately grades gliomas and holds potential for clinical application in the field of medical diagnostics.

## Figures and Tables

**Table 1:** Results of evaluation metrics for different modalities under 2D CNN (A) and 2D CNN with 3D features (B). Using the results obtained for each modality under 2D CNN as a baseline. Evaluation indicators include AUC (Area Under Curve), ACC (Accuracy), APCA (Average Per-Class Accuracy), SEN (Sensitivity) and SPE (Specificity).

(A)						
		**2D**				
		**AUC**	Acc	APCA	SEN	SPE
**single-modal**	T1	0.6451	0.7226	0.6218	0.8482	0.3953
	T2	0.9178	0.8774	0.8149	0.9554	0.6744
	T1-Gd	0.9120	0.8194	0.7580	0.9604	0.5556
	FLAIR	0.9079	0.8452	0.7787	0.9533	0.6042
**multi-modal**	**T1+T2+T1-Gd+FLAIR**	0.9565	0.8774	0.8171	0.9811	0.6531
(B)						
		**2D+3d_features**				
		**AUC**	Acc	APCA	SEN	SPE
**single-modal**	T1	0.8284	0.8129	0.7396	0.9259	0.5532
	T2	0.9499	0.9097	0.8558	0.9735	0.7381
	T1-Gd	0.9317	0.8839	0.8239	0.9478	0.7000
	FLAIR	0.9227	0.8903	0.8352	0.9407	0.7297
**multi-modal**	**T1+T2+T1-Gd+FLAIR**	0.9684	0.9936	0.9853	1.0000	0.9706

**Table 2:** Results of evaluation metrics for different modalities under 3D CNN.

		3D				
		AUC	Acc	APCA	SEN	SPE
**single-modal**	T1	0.6631	0.7871	0.6792	0.8385	0.5200
	T2	0.7321	0.6194	0.6561	0.9559	0.3563
	T1-Gd	0.8250	0.7742	0.7061	0.9216	0.4906
	FLAIR	0.7336	0.7161	0.6667	0.9140	0.4194
**multi-modal**	**T1+T2+T1-Gd+FLAIR**	0.6626	0.6129	0.6075	0.8861	0.3289

**Table 3:** AUC results of ablation experiments using 2D CNN with 3D features model for different data augmentation strategies.

Strategy	AUC
w/o Augment	0.9492
w/o CyclicShift	0.9582
w/o Rotation	0.9628
w/o Flip	0.9638
w/o Duplication	0.9480
Our work	0.9684
